# News and Events of the Week

**Published:** 1910-10-29

**Authors:** 


### News and Events of the Week.

The new clinical medicine laboratory which has been installed in the Royal Infirmary of Edinburgh through the generosity of an anonymous friend of Dr. George A. Gibson, of Edinburgh, was opened last week by Sir Clifford Allbutt, Regius Professor of Physic at Cambridge. Professor Rankine presided, and Sir Clifford Allbutt said that it was one step more towards the realisation of the integration of pathological research and clinical medicine which had been unfortunately separated in the location of laboratories and hospitals.

The Royal Society of Medicine (Odontological Section) has issued the regulations for grants in aid of Scientific Research, from which it appears that all grants expire on May 1 next following the date on which they are made, when a statement of expenditure shall be forwarded to the Hon. Secretary. No part of any grant shall be applied in payment of personal expenses in connection with the research for which the grant has been made, unless a resolution to that effect has been duly passed by the Committee. Every recipient of a grant shall be deemed to have notice of these rules.

A recent meeting at the St. Marylebone General Dispensary inaugurated the Society of Infant Consultations. The chair was taken by Dr. Wynter Blyth. The Chairman, Dr. Eric Pritchard, and Dr. Sykes explained the advantages of infant consultations in combating infantile mortality. Hopes were expressed that lay workers who were in any way connected with infant consultations would join the Society. The Hon. Secretaries are Dr. Ronald Carter, 11 Leonard Place, Kensington, W., or Dr. Janet Lane-Claypon, 69 Prince of Wales Mansions, Battersea Park, S.W.

We take the following plea for a London motor-ambulance service from a recent letter of Sir William Collins' in the *Times*: "By the Metropolitan Ambulances Act, which I was fortunate in piloting through Parliament last session, the London County Council is now in a position to establish and maintain an efficient service for the county like that which has worked so well in the City. The Home Secretary moreover informed me, in reply to a question, that he was ready to sanction any practicable arrangement for the co-operation of the police with the Council in carrying out such improved service. When the new County Council was elected last March it was understood that at least a majority of its members were pledged to put into action the powers which the Council had possessed since October 1909; but as yet I cannot learn that any effective steps have been taken."

Dr. John Thomas Faulkner, of Apsley House, Chester Road, Stretford, Manchester, who died on August 6, aged fifty-four, has left estate valued at £33,083 gross and £11,556 net.

Professor Richard Berry, M.D. (Victoria), has been elected a Fellow of the Royal Colonial Institute, and Mr. R. Fleming Jones, M.D., Samarai, Papua, has been appointed an Honorary Corresponding Secretary.

Lieut.-Col. C. H. Melville, Professor of Hygiene at the Royal Army Medical College, is delivering a series of six lectures at the Royal Sanitary Institute on Army Sanitation. These lectures are instituted principally for sanitation officers of the Territorial Forces.

A book on "English Philosophy," from the pen of Mr. Thomas M. Forsyth, of Edinburgh, is shortly to be issued by Messrs. A. and C. Black. Its aim is to give an outline of the development of English Philosophy, from Bacon to the present day. Finding the nature of the development in the progressive employment of the English method (known distinctly as the "method of experience") it endeavours to show how advance in method and in results have accompanied and conditioned each other. Among the phases of philosophy embraced by Dr. Forsyth's comprehensive volume there will be the inauguration of the Experimental Philosophy in Bacon; the modification of the Baconian method employed by Hobbes in the construction of a system of doctrine; the development from Locke to Hume, and many other chapters leading up to what may be called experimentalism.

Dr. Purves Stewart gave the opening address of the winter session at the Central London Throat and Ear Hospital on Monday, October 17, taking for his subject "Certain Intra-cranial Diseases Associated with Nasal, Aural, and Laryngeal Symptoms." The address, which was illustrated by lantern slides, attracted a crowded audience. On the same evening the annual dinner of the medical staff, together with past and present post-graduate students of the hospital, took place at the Trocadero Restaurant, Mr. Stuart-Low in the chair. The consulting surgeon and consulting physician, Sir Watson Cheyne and Dr. Purves Stewart were present, together with every member of the active staff, whilst among the guests were Sir John Young, Mr. James Berry, Mr. Mayo Collier, Mr. Betham Robinson, Major Vint, Dr. Kelson, Dr. Drummond Morier, Mr. Creasy, Mr. Charles Heath, Mr. Work Dodd, Dr. Wiltshire, Mr. Claud Woakes, Dr. Sim Wallace, Dr. Vaughan Jackson, Dr. Napier Grant, Mr. Chaldecott, Dr. Forsyth, etc. The toast of "Success to the Hospital" was proposed by Mr. Charles Heath.

The studentship on the foundation of the late Professor Tyndall for scientific research on subjects tending to improve the conditions to which miners are subject has been awarded by the Royal Society for the ensuing year to Dr. T. L. Llewellyn, of Bargoed, Wales, for research regarding the cause and cure of nystagmus in miners.

---

Alderman Sir Thomas Crosby, M.D., who was chairman of the committee appointed to arrange for the reception given by the Corporation to the British Medical Association in the Guildhall in July, has been presented by the committee with a silver centrepiece as a souvenir of the occasion from the hands of the Lord Mayor.

---

Dr. G. W. Willoughby, of Eastbourne, has been installed President for the coming year of the Society of Medical Officers of Health. The Society was founded in 1856, and has now over one thousand members, many of whom attended the annual reception and dinner of the Society, which took place last week. Sir Dyce Duckworth and Mr. H. T. Butlin were among the guests.

---

Miss E. M. Magill has been appointed to inspect girls in the Essex county secondary schools. The appointment is made in accordance with a recent decision of the Essex Education Committee that medical inspection should be extended to all children attending county secondary schools. Authority was then given to a special committee to appoint a woman doctor for this purpose.

---

The first degree ceremony in connection with the University of Bristol took place on October 20 in the Victoria Rooms. The Vice-Chancellor (Sir Isambard Owen) presided, and among those present were the pro-Vice-Chancellor, Professor Michell-Clarke, the Lord Mayor and Sheriff, members of the Corporation and Council, members of the University Council and staff, members of the Court of the University, the Dean and Chapter, Magistrates, graduates, and others. The presenters were Deans of the several faculties. In the Faculty of Arts, Professor Brooks, M.A.; in the Faculty of Science, Professor Barrell, M.A.; in the Faculty of Medicine, Professor Fawcett, M.D., C.M.; in the Faculty of Engineering, Professor Wertheimer, B.A., B.Sc. The honorary degree of Doctor of Science was conferred on Professor Conwy Lloyd Morgan, LL.D., F.R.S. The hood of the graduates of the University is of a special tinge of red, termed University red. It is taken from the general colour of the rocks and earth in the neighbourhood of Bristol. The University undergraduates indulged in a "rag," and marched in procession back to the University. They gave good evidence as to the soundness of their lungs.

---

The Board of Education has addressed a letter to the London County Council, stating that as a result of its observation of the actual working of medical inspection in the London schools, it is informed that the work of the inspecting officers is generally thorough; but that (1) the staff of inspecting officers is not adequately organised and supervised; (2) the arrangements for submitting scholars to inspection required by the Act of 1907 have not been complied with; (3) by adopting a scheme which differs widely from those of other great educational authorities the Council has failed to put the work of amelioration on a sound basis; (4) even the proposals for strengthening the medical staff, recommended for adoption from January 1, 1911, are insufficient; and (5) that an early and precise reply is asked to these criticisms, stating what changes are to be brought about during the present autumn.

The Röntgen Society, founded in 1897, begins its fourteenth session on November 3, at 20 Hanover Square. The Society, the parent Society of the Electro-Therapeutic Section of the Royal Society of Medicine, is in a flourishing condition, as the syllabus for the coming session shows. So much sensational literature on radium has been published in the public Press that the lecture by Professor Rutherford at the Röntgen Society will be listened to with much advantage. Professor Rutherford, who is the greatest authority in Great Britain on radium, has the unique advantage of possessing more radium than anyone else in the British Isles. The price of radium at the present time is about £224,000 per oz., and the price of small quantities is difficult to estimate, but the Röntgen Society possesses three carefully prepared standards by which the value of unknown quantities of radium can be compared and their value estimated. Professor Soddy, of Glasgow, will also lecture at the Society. His work is well known in connection with radium and in conjunction with Sir William Ramsay's and Sir James Dewar's work on helium. From Amsterdam will come a great physicist in the person of Professor Salomonson, who will unfold some original work on the mysteries of the working of an induction coil by which $x$-rays are produced. Some most original work on $x$-rays has been done by Professor Barkla, of King's College, and he will describe his results at a lecture. Professor Barkla's work formed the foundation of a lecture delivered by Sir J. J. Thompson at the British Medical Association this year. Dr. Hall Edwards, who has now freed himself from his former suffering, caused by $x$-rays, has also promised to take part. Other well-known scientists will also lecture. These lectures are published in the Journal of the Röntgen Society. Invitations and further information can be had from the Hon. Secretary, Dr. A. Howard Pirie, 7 Harley Street, W.

